# Supplementary material for: Microfluidic-Assisted Silk Nanoparticles Co-Loaded with Epirubicin and Copper Sulphide: A Synergistic Photothermal–Photodynamic Chemotherapy Against Breast Cancer
Source: Nanomaterials (Basel). 2025 Jan 30;15(3):221. doi: 10.3390/nano15030221 (PMC11820260; doi:10.3390/nano15030221)
Supplement: Supplementary file 1 [file nanomaterials-15-00221-s001.zip › nanomaterials-3446354-supplementary.pdf]

nanomaterials

Article

Microfluidic-Assisted Silk Nanoparticles Co-Loaded with Epirubicin and Copper Sulphide: A Synergistic Photothermal-Photodynamic Chemotherapy Against Breast Cancer

Zijian Gao [1*], Muhamad Hawari Mansor [1*], Faith Howard [1], Jordan MacInnes [2], Xiubo Zhao [3,], and Munitta Muthana [1,**]**

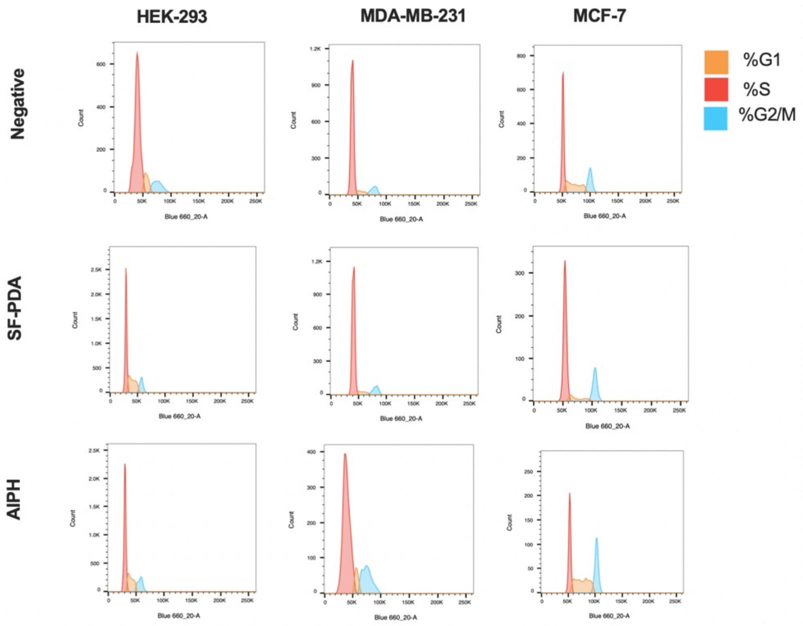

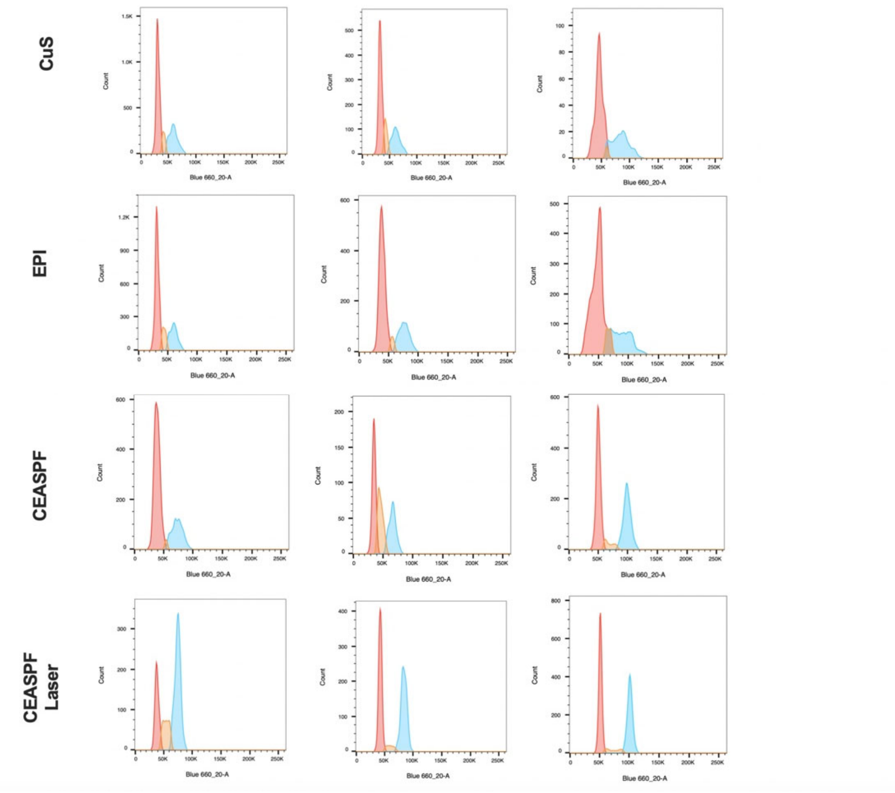

Figure S1 NIR light activation prompts designed CEASPF NPs to disrupt cancer cell growth by specifically interfering with the G2/M phase of the cell cycle. Flow cytometry cell cycle analysis of MDA-MB-231, MCF-7 breast cancer cells, and HEK-293 human embryonic kidney cells treated with negative control and 100 μg/mL of SF-PDA, CEASPF (CuS-EPI-AIPH@SF-PDA-FA with and without NIR irradiation) and free AIPH, CuS, and EPI for 24 h of incubation. Error bars are hidden in the bar when not visible; data are mean ±SD, n ≥ 3. (* $p < 0.03$, ** $p < 0.002$, *** $p < 0.0002$, **** $p < 0.0001$).

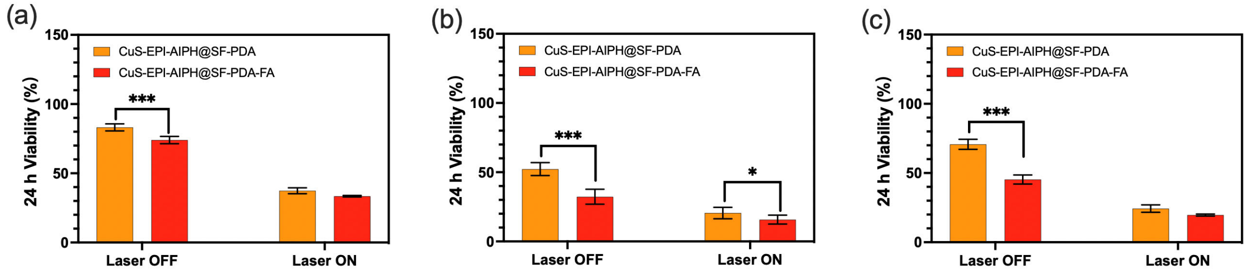

Figure S2 The cytotoxicity of 100 μg/mL CuS-EPI-AIPH@SF-PDA and CuS-EPI-AIPH@SF-PDA-FA nanoparticles was assessed in (a) HEK-293 human embryonic kidney cells, (b) MCF-7 human breast cancer cells, and (c) MDA-MB-231 human breast cancer cells after 24 hours of treatment, with and without 808 nm NIR exposure for 5 minutes at an intensity of 2 W/cm². Error bars are not visible when hidden in the bar; data are presented as mean ± SD, with n ≥ 3. (*$p < 0.03$, **$p < 0.002$, ***$p < 0.0002$, ****$p < 0.0001$).

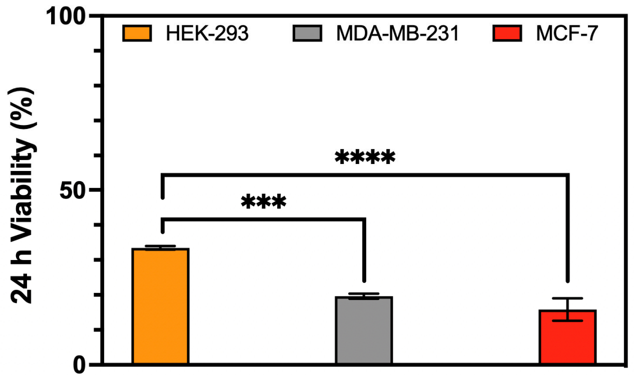

Figure S3 The cytotoxicity of 100 μg/mL CuS-EPI-AIPH@SF-PDA-FA nanoparticles was assessed in HEK-293 human embryonic kidney cells, MDA-MB-231 human breast cancer cells, and MCF-7 human breast cancer cells after 24 hours of treatment, with 808 nm NIR exposure for 5 minutes at an intensity of 2 W/cm^2. Error bars are not visible when hidden in the bar; data are presented as mean ± SD, with $n \geq 3$. (*$p < 0.03$, **$p < 0.002$, ***$p < 0.0002$, ****$p < 0.0001$).
